# Capture silk scaffold production in the cribellar web spider

**DOI:** 10.1186/s42649-021-00061-y

**Published:** 2021-07-13

**Authors:** Yan Sun, Seung-Min Lee, Bon-Jin Ku, Eun-Ah Park, Myung-Jin Moon

**Affiliations:** Department of Biological Sciences, Dankook University, 31116 Cheonan, Korea

**Keywords:** Cribellum, Scaffold, Silk, Spider, Spigot

## Abstract

Spider capture silk is a natural scaffolding material that outperforms most synthetic materials in terms of its combination of strength and elasticity. Among the various kinds of silk threads, cribellar thread is the most primitive prey-capturing type of spider web material. We analyzed the functional organization of the sieve-like cribellum spigots and specialized calamistral comb bristles for capture thread production by the titanoecid spider *Nurscia albofasciata*. The outer cribellar surface is covered with thousands of tiny spigots, and the cribellar plate produces non-sticky threads composed of thousands of fine nanofibers. *N. albofasciata* cribellar spigots are typically about 10 μm long, and each spigot appears as a long individual shaft with a pagoda-like tiered tip. The five distinct segments comprising each spigot is a defining characteristic of this spider. This segmented and flexible structure not only allows for spigots to bend individually and join with adjacent spigots, but it also enables spigots to draw the silk fibrils from their cribella with rows of calamistral leg bristles to form cribellar prey-capture threads.

## Introduction

Araneomorph spiders can be classified as cribellate or ecribellate based on the presence or absence, respectively, of a cribellum in addition to multiple spinnerets (Coddington and Levy 1991). The cribellum is a silk-spinning organ consisting of single or multiple plates covered by thousands of minute spigots, which produce fine fibrils that are quickly hackled by the calamistrum, producing woolly-structured silk (Foelix 2011).

Cribellar silk has dry-adhesive properties and originates from cribellar fibrils spun from cribellar spigots (Opell 1995, 1999). The fibers are extremely fine, which facilitates prey entanglement, disengagement, and capture without the need for adhesive substances (Opell 2002; Hawthorn and Opell 2003).

Cribellate web production is considered an expensive capture technique because of the extensive labor required to comb and deposit cribellar silk (Opell et al. 2000; Opell and Schwend 2009). However, cribellar silk improves is advantageous because it facilitates prey retention rather than delaying prey passage (Opell 2002). Cribellar silk production and prey wrapping are similar in terms of the composition of the output material, and this suggests that, as a source of dense silk, the cribellum may be an evolutionary adaption originating from the anterior median spinnerets (Opell et al. 2011).

Given that the number of cribellar spigots corresponds with cribellar thread stickiness (Opell 2002; Opell and Schwend 2009), cribellar spinning plate morphology and spinning apparatus distribution are key characteristics of cribellate spiders (Park and Moon 2009). The basic principles of the cribellate spinning process were recently delineated using *Uloborus plumipes* as a representative species; cribellar fibers were demonstrated to be organized in the form of a mat surrounded by paired axial fibers. Furthermore, *Zosis geniculata* capture threads were observed to have interconnections between the cribellar mat and axial fibers (Joel et al. 2016).

A calamistrum is a row of specialized appendages, or bristles, that spiders use to comb out fine silk fibril bands (Foelix 2011). Certain spiders use such a comb-like apparatus to split the silk fibers drawn from the cribellum into finer fibers, giving it a woolly structure. Spiders use the calamistrum and cribellum to form the hackled silk bands that characterize cribellate spider webs (Opell et al. 2000; Joel et al. 2016). While the cribellum is an oval spinning field with spigots that produce the silk fibrils forming the outer surface of the primitive prey-capture threads of aerial spider webs (Opell 1999), the calamistrum pulls cribellar silk fibrils and facilitates their combination with supporting strands to form cribellar prey-capture threads (Opell et al. 2000).

Recent research has revealed that the fibers from cribellate spinning spigots pass through a smooth surface-like region on the calamistrum. Kronenberger and Vollrath (2015) demonstrated that dry capture threads combine thousands of single nanoscale filaments issuing individually from single spigots to be electrically charged by special limb combs. In particular, the contact between the fibers and the calamistrum can be adjusted after thread production without modifying the calamistrum’s influence on the fibers (Joel et al. 2016). However, it is not clear how cribellar nanofibers can effectively assemble a puffy structure within a capture thread using the specialized calamistral setae.

*Nurscia albofasciata* is a cribellate spider species that produces silk webs with the aid of calamistra. *N. albofasciata* is among the most abundant and conspicuous spiders in temperate regions, but not much is known about their cribella and calamistra. Thus, this article describes the functional organization of the cribellar spigots and calamistra of the titanoecid spider *N. albofasciata* through field emission scanning electron microscopy (FESEM).

## Materials and methods

Adult individuals of the cribellate spiders of the family Titanoecidae (Araneae: Titanoecidae) were collected in a local area near the dumping site of dredging soil at the Busan New Port of Jinhae city, Kyungnam, Korea. All spiders were maintained under ambient conditions with natural lighting in enclosures comprising a wooden cage. They were fed insects and water daily.

Both of female and male specimens were anesthetized with CO_2_ and dissected under a dissecting light microscope in a drop of spider Ringer’s solution consisting of 160 mM NaCl, 7.5 mM KCl, 4 mM CaCl_2_, 1 mM MgCl_2_, 4 mM NaHCO_3_ and 20 mM glucose, pH 7.4 (Moon and Tillinghast 2020). The specimens for light microscopic preparation were fixed in alcoholic Bouin’s solution, embedded with Paraplast embedding medium (Fisher Scientific Co., Pittsburgh, Pa, USA) and stained with hematoxylin and eosin solution.

For field emission scanning electron microscopy, the whole abdomen was gently removed and pre-fixed in a mixture of 2 % paraformaldehyde and 2.5 % glutaraldehyde buffered with 0.1 M phosphate buffer at pH 7.4 for 2 h. Postfixation was performed with 1 % osmium tetroxide in the same buffer and washed several times in 0.1 M phosphate buffer for 1 h. After each fixation step, the samples were rinsed three times with phosphate buffered solution at 15 min intervals. The samples were then dehydrated through a graded series of ethanol from 50 % to absolute ethanol, and then transferred to hexamethyldisilazane (HMDS) for air-dry (Seo et al. 2020).

The samples were then coated with platinum-palladium with a thickness of 20 nm, using a Hitachi E-1030 ion sputter coater (Hitachi Co., Tokyo, Japan). Coated samples were observed with a Hitachi S-4300 (Hitachi Co., Tokyo, Japan) field emission scanning electron microscopy (FESEM) with an accelerating voltage of 5–20 kV.

## Results

**Fig. 1:**
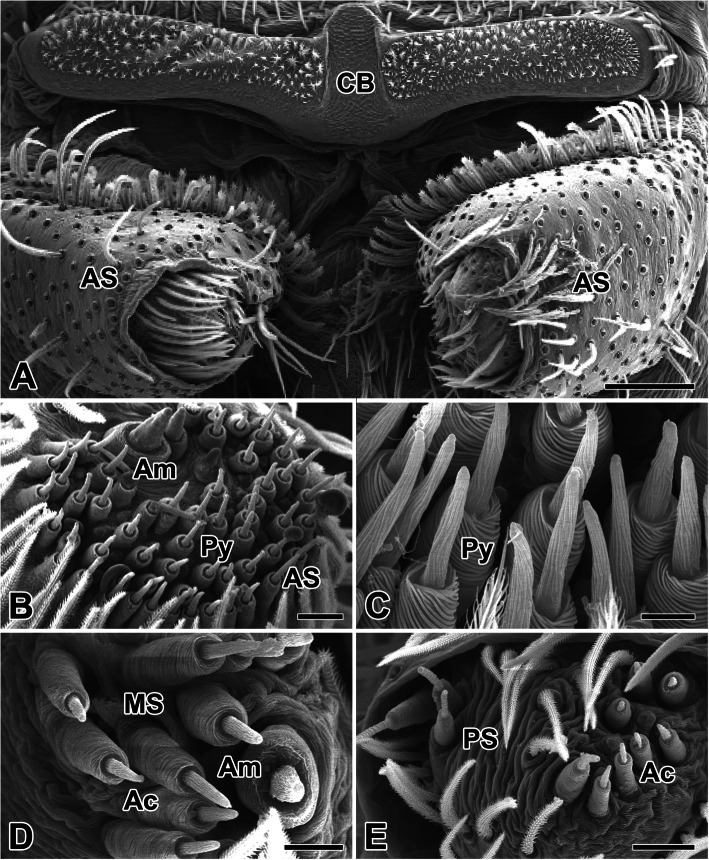
Scanning electron micrographs of the cribellum and spinnerets in the spider *N. albofasciata*. **a** Cribellum (CB) is located at the upper region of the anterior spinnerets (AS). **b, c** On the anterior lateral spinneret, the major ampullate gland (Am) and the pyriform gland (Py) spigots were observed. **d** On the posterior median spinneret (MS) the minor ampullate gland and the aciniform gland (Ac) spigots were distributed. **e** On the posterior lateral spinnerets (PS), spigots of the aciniform glands are seen. Scale bars indicate 100 μm (**a**), 20 μm (**b, e**), 10 μm (**d**), and 5 μm (**c**), respectively

In *N. albofasciata*, the cribellum is located on the ventral abdominal surface, at the upper region of the spinnerets. Although the term cribellum literally means “little sieve” and applies to biological structures in the form of tiny, perforated plates, this broad plate is set firmly in the abdominal cuticle. The cribellar spinning field is divided longitudinally into two well-defined halves (Fig. 1a).

Apart from the cribellar spigots, we observed three pairs of spinnerets in *N. albofasciata*. Two types of silk spigots the major ampullate gland and the pyriform gland spigots were distributed on the anterior lateral spinneret (Fig. 1b, c). Two types of spigots the minor ampullate gland and the aciniform gland spigot were observed on the posterior median spinneret (Fig. 1d). *N. albofasciata* spiders also had aciniform gland spigots on their posterior lateral spinnerets (Fig. 1e).

**Fig. 2:**
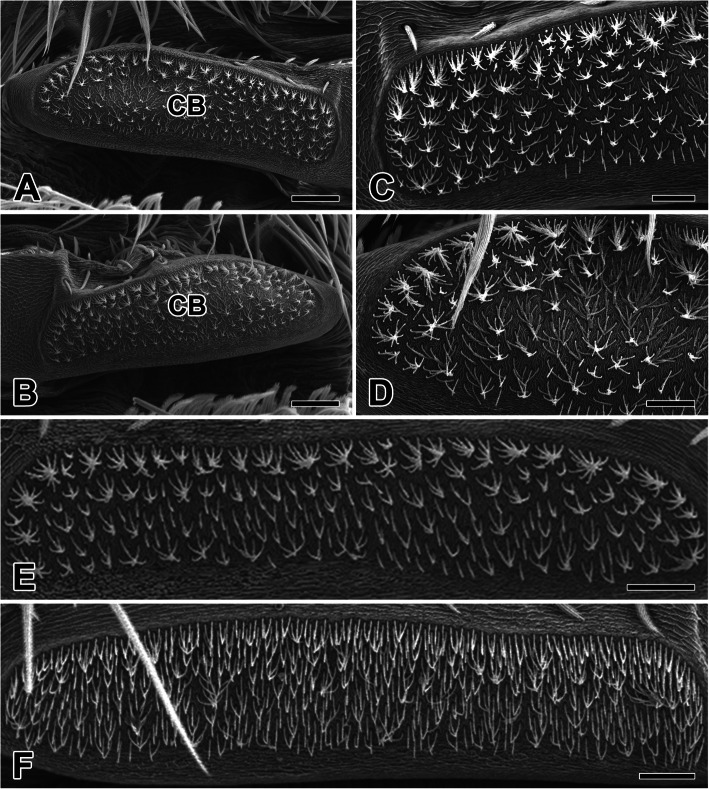
Scanning electron micrographs of the cribellum in the spider *N. albofasciata*. **a, b** The cribellar silk spinning system consists of bipartite cribellum (CB) plate which divided with left and right spinning fields. **c, d** A pair of cribellar plate with hundreds to thousands outlets are medially divided to form a symmetrical distribution of spinning apparatuses. **e, f** The surface of the cribellum is covered by numerous elongate spigots which producing numerous cribellate silk fibrils. Scale bars indicate 50 μm (**a, b**) and 20 μm (**c-f**)

*N. albofasciata* spiders possess bipartite cribellar plates, which divided the left and right spinning fields (Fig. 2a, b). The cribellar surface is covered with thousands of elongate spigots, all acting together to produce cribellar threads composed of thousands of silk fibrils (Fig. 2c, d). The total number of cribellar spigots varies between spiders according to their maturity, with male spiders averaging about 500 pairs and females averaging about 800 pairs (Fig. 2e, f).

**Fig. 3:**
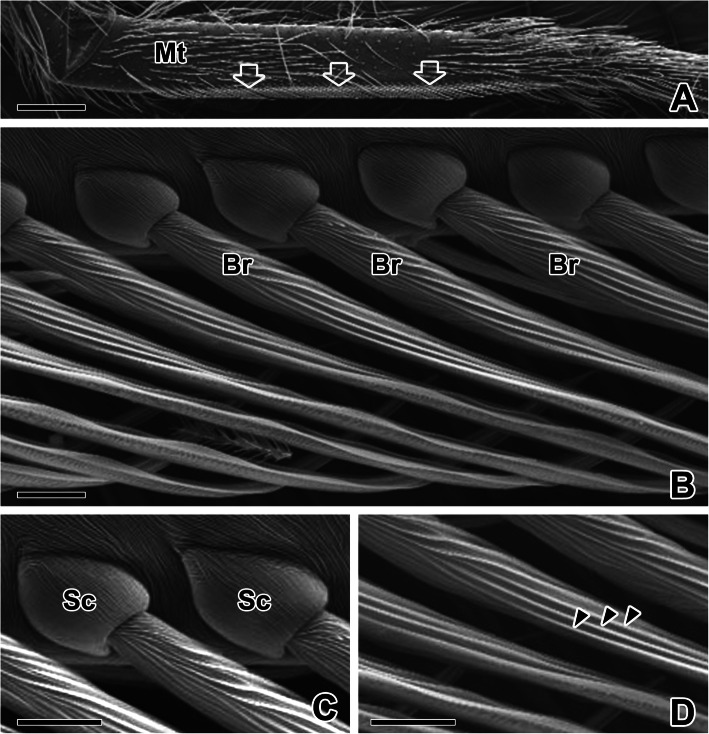
Scanning electron micrographs of the calamistrum combs in the spider *N. albofasciata*. **a** On the upper margin of the metatarsus (Mt) segment of the 4th leg, a specialized comb of bristles, called a calamistrum (arrows) is located. **b** The bristles (Br) are long, straight, and have a pointed end toward the tip. **c** Each bristle of the calamistrum is embedded in a cuticular socket (Sc). **d** The surface of the bristle is completely covered with longitudinal grooves (arrowheads). Scale bars indicate 200 μm (**a**) and 10 μm (**b-d**)

This cribellate spider also has calamistra that comb the silk that flows from the cribellum, producing characteristically woolly silk. A specialized comb of bristles is located only on each leg of the last pair of legs among four pairs of limbs. The calamistrum is found on the upper metatarsal margin (Fig. 3a). The bristles are used to simultaneously comb out masses of cribellar fibrils and their supporting silk lines from the cribellum (Fig. 3b). Each calamistral bristle is embedded in a cuticular socket, and each is serrated on one side and smooth on the other. The setaceous surfaces are completely covered with grooves (Fig. 3c). The bristles are strong and mostly long and straight but some are partly sickle-shaped with pointed, longitudinal grooves toward the ends. Each bristle is in an open articulatory socket, and the gap between each bristle is approximately 5 μm wide (Fig. 3d).

**Fig. 4:**
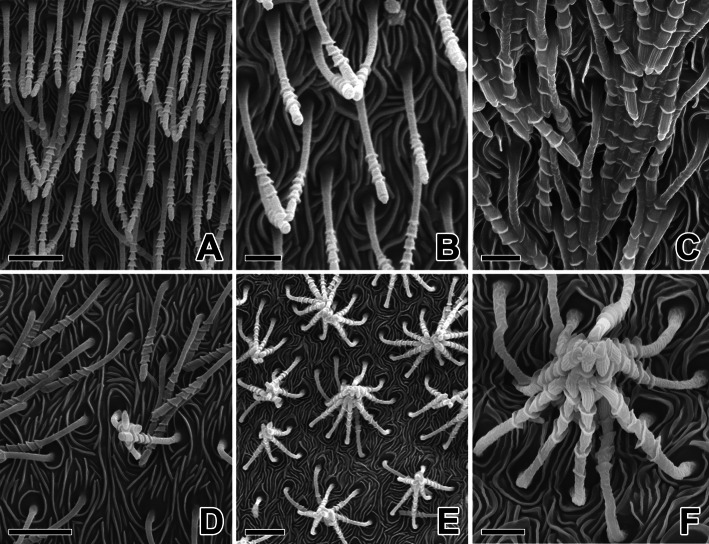
Scanning electron micrographs of the cribellate spigots in *N. albofasciata*. **a-c** The cribellate spigots appeared as singular, long shafts with pagoda-like tiered tips. **d-f** All spigots are all approximately the same length (10 μm). These segmented and flexible structure enable to bent itself and conjoin together with adjacent other spigots. Scale bars indicate 5 μm (**a, d, e**) and 2 μm (**b, c, f**)

The cribellar silk in *N. albofasciata* is produced from the cribellar spigots, which appear as individual, long shafts with pagoda-like tiered tips. The cribellar silk is produced through these elongate spigots, which protrude from the cribellar plates (Fig. 4a-c). All spigots act together to simultaneously produce numerous cribellar silk fibrils. All of the cribellar spigots are approximately 10 μm long. Their segmented and flexible structure enables cribellar spigots to bend individually and functionally combine with adjacent spigots (Fig. 4d-f).

**Fig. 5:**
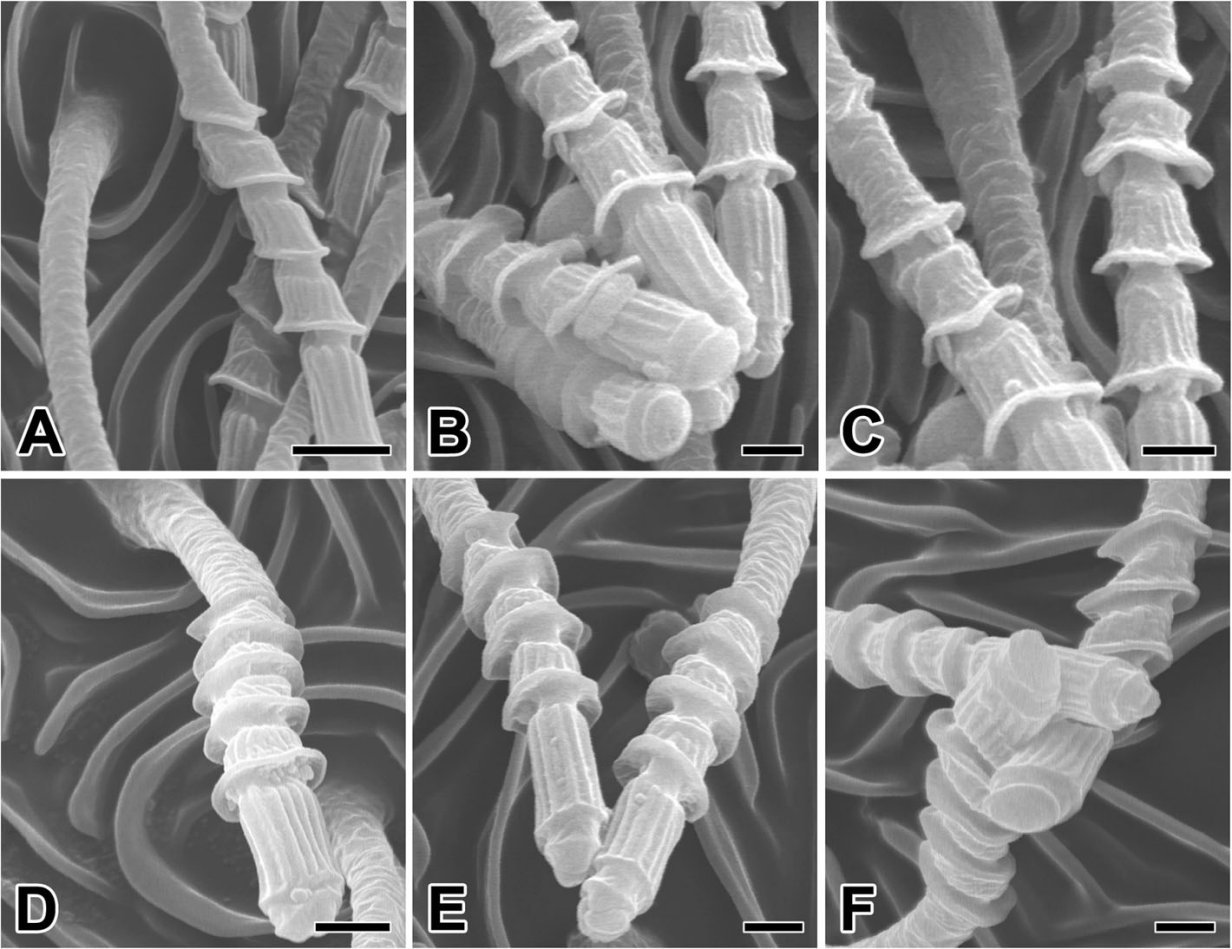
High magnification scanning electron micrographs of the cribellate spigots in *N. albofasciata*. **a-c** Cuticular surface of the cribellum is covered by hundreds or thousands of tiny, elongate spigots, each producing a single fibril of cribellate silk. **d-f** All of these spigots are composed of five tubal segments with four thicker regions. Each cribellar spigot shows segmented flexible structure which enable to bent itself and conjoin together with adjacent spigots. Scale bars indicate 1 μm (**a**) and 0.5 μm (**b-f**)

All of the *N. albofasciata* cribellar spigots are composed of five tubal segments with four thicker regions that provide the characteristic pearling node of cribellar threads (Fig. 5a-c). The cribellar silk-spinning system contains tiny silk glands, each terminating through long and narrow ducts. The cribellar plate is composed of thousands of spinning outlets, with larger spiders having more outlets (Fig. 5d-f).

**Fig. 6:**
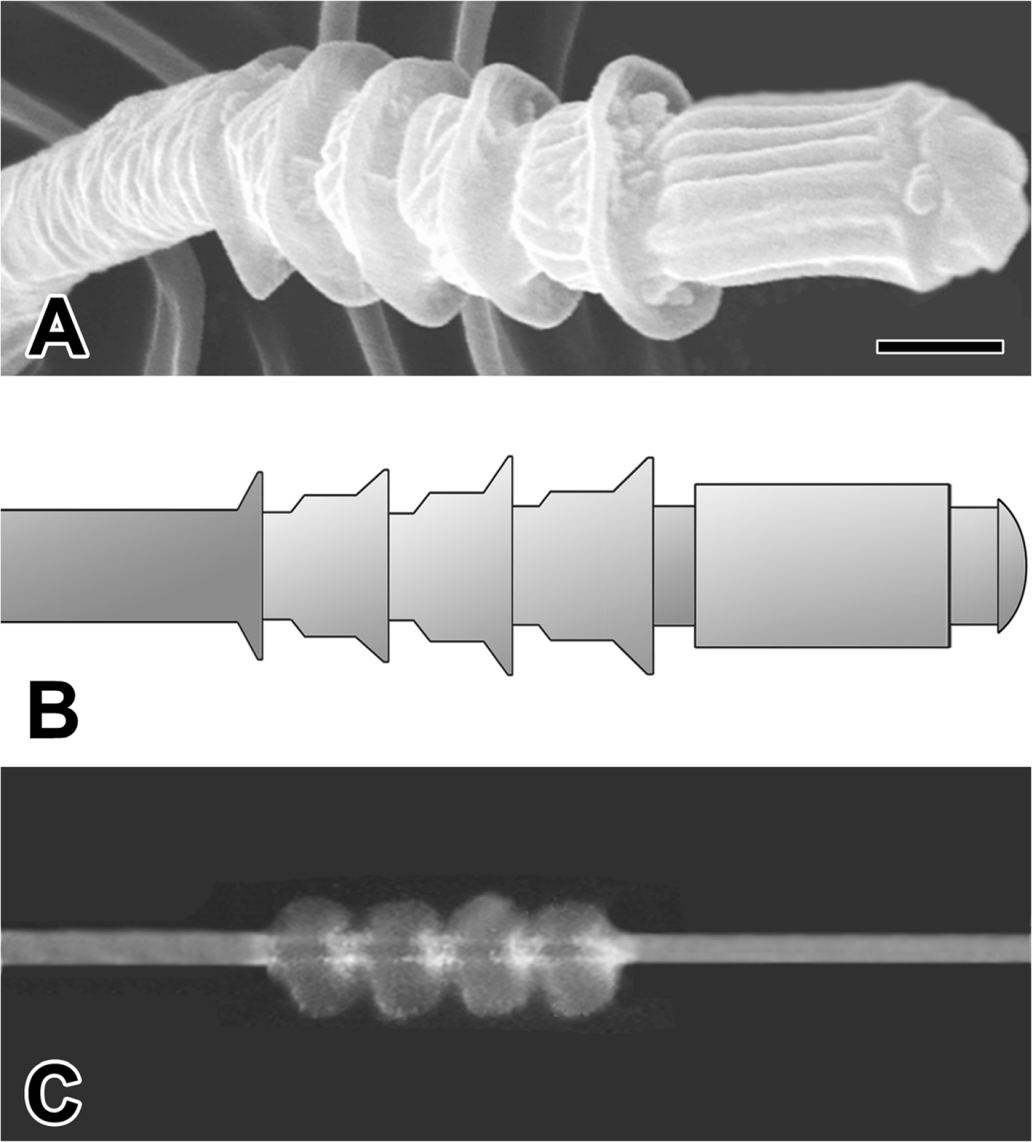
**a** Scanning electron micrograph of the cribellate spigots in *N. albofasciata*. The cribellate spigots are composed of five tubal segments with four thicker regions which will provide the characteristic pearling node of the cribellate threads. **b** Diagram of the cribellate spigot which composed of five tubal segments with for thicker regions. **c** Photo micrograph of the cribellate silk line. Each silk line has a unique outer morphology resembling the segmented insulators since the silk filaments are spun out from the spigot by the rapid hackling. Scale bar indicates 0.5 μm

The expanded intersegmental spaces finally give rise to pearling of the cribellar thread and provide supporting points to hold silk fibrils during the hackling process carried out by the calamistra (Fig. 6a, b). Thus, the calamistral leg bristles draw cribellar silk fibrils and facilitate their combination with supporting strands to form cribellar prey-capture threads (Fig. 6c).

## Discussion

Spiders can be classified according to their shape and the components of their silk-spinning apparatuses. Although these apparatuses characterized by adaptative variations, some basic features remain unchanged at the familial phylogenetic level (Peters 1987; Shear 1994). Previous researchers have found that silk-spinning apparatuses are characterized by functional specialization involving precise spinneret modifications, anatomical silk glands characteristics, and spigot number and morphology (Peters and Kovoor 1991; Moon and Tillinghast 2004; Foelix 2011; Park and Moon 2014).

Spiders produce various silk types for a remarkably diverse array of specific tasks (Denny 1976; Coddington 1986), but catching prey is the main function of spider silk (Nentwig and Heimer 1987). Spider and their webs can be classified as cribellate or ecribellate according to prey catching systems (Foelix 2011). Ecribellate spiders rely on a wet glue spinning process that uses a liquid silk solution to form aqueous droplets on core filaments (Vollrath and Knight 2001; Park and Moon 2014). Cribellate spiders, on the other hand, produce dry capture threads from a cribellum (Peters 1987; Peters and Kovoor 1991; Bott et al. 2017).

Most spiders produce silk fibers with diameters on the micrometer scale, but cribellate spiders spin nanoscopic fibers. Recently, Kronenberger and Vollrath (2015) showed that the nano-scale fibers spun by the cribellate orb spider, *U. plumipes*, are electrically charged to facilitate prey capture. They hypothesized that, *U. plumipes* cribellar spigots have unique morphological features, with an outer bearing a striking resemblance to the multilayered ‘weather sheds’ design of high-voltage insulators for preventing flow via leakage (Suwarno 2009; Kronenberger and Vollrath 2015).

Previously, Opell and Schwend (2009) and Opell et al. (2011) also revealed that cribellar silk captures and holds prey via van der Waals interactions, including with the involvement of longer-range electrostatic forces. We also observed that the cribellar structure, including the long and slender cribellar spigots and the hind-leg calamistra was likely associated with electrostatic charging during the spinning of nanoscale fibers.

*N. albofasciata* cribellar silk originates from abdominal spigots situated on the pair of medially divided plates that from the cribellum. These specialized anatomical features are characteristic of all cribellate spiders with divided cribellar spinning plates (Opell 2002; Opell et al. 2011; Hajer et al. 2017). Fine structural analyses using scanning electron microscopy, have revealed that the cribellar surface is covered by hundreds or thousands of tiny, elongate spigots, each producing solitary nanoscopic cribellar silk fibrils.

Cribellar threads are primitive prey-capture filaments formed from thousands of fine, looped cribellar fibrils. This means that, the number of cribellar spigots correlates with cribellar thread stickiness (Opell et al. 2011; Hajer et al. 2017). Opell (2002) showed that the linear cribellar thread spun from divided *Kukulcania hibernalis* cribellum was both wider and stickier than thread from undivided *Waitkera waitakerensis* cribellum. Since the divided *K. hibernalis* cribellum and the undivided *W. waitakerensis* cribellum had similar spigots counts and produced cribellar threads with similar stickiness, the spinning anatomy and spinning behavior of both spiders affect cribellar thread stickiness.

It is likely that a dry web made with such a composite wool-like thread meshwork is particularly effective at tangling the claws, bristles, and spines of insect prey. The fine cribellar silk fibrils are also dry adhesives with electrostatic properties and can even adhere to smooth beetle cuticle (Opell 2002). Previous studies have demonstrated at least two major stickiness mechanisms on which cribellar thread appears to rely. Surface fibrils can snag on the setae of a prey insect holding them like the looped surface ace of a Velcro fastener (Autumn et al. 2000; Hawthorn and Opell 2003). Via unknown mechanisms, cribellar thread has also been known to cling to smooth surface, with no protrusions for snagging (even on a microscopic level), such as graphite, polished steel, and glass (Eberhard 1980, 1993). Cribellar thread adheres more tightly to smooth beetle elytra than to the heavily setose fly nota (Opell and Schwend 2009).

Scanning electron microscopy, has shown *N. albofasciata* to possess paired calamistra, each with a row of toothed bristles on the metatarsal segments of the respective hind legs. Spiders use these bristles to simultaneously comb out cribellar fibrils and their supporting silk lines from the cribellum and spinnerets. Therefore, silk fibrils are spun collectively by the comb hairs on the spider’s hind legs and jerked out of their spigots by rapid hackling.

Recently, Kronenberger and Vollrath (2015) demonstrated that the fiber-forming process used by *U. plumipes* (a cribellate orb spider) is different from the silk-spinning systems of all other known spiders. *U. plumipees* cribellar glands have long ducts but lack the internal extrusion process draw-down. To flow in the spigot pockets, the viscosity of the dope for the cribellar silk must be exceptionally low, and it must be liquid through to the spigot because when the silk reaches the spigot, it is already in thread form (Vollrath and Knight 2001; Davies et al. 2013).

The authors suggest that it takes milliseconds for the silk to solidify between each violent hackling pull before being ‘frozen’ into shape during the pulling post-draw. Their electron microscopy examination of the cribellar glands ducts revealed this interpretation, which was corroborated by our separate analysis of *N. albofasciata* in which we observed specialized pearling chambers, among the spigots, which filled with silk material.

The cribellar silk-spinning system in *N. albofasciata* is composed of tiny silk glands each terminating through exceptionally long and narrow ducts. Additionally, each cribellar spigot is segmented and flexible, which enables the spigots to bend individually and functionally combine with one another. In particular, all of the spigots are composed of five tubal segments with four thicker regions that enable the pearling node of the cribellar threads. This expanded intersegmental space finally yields pearling of the cribellar thread and provides supporting points to hold silk fibrils during the hackling process performed by the calamistral leg combs. Thus, calamistra draw silk fibrils from the cribellum and help combine the fibrils with supporting strands to form cribellar prey-capture thread.

## Conclusions

We investigated the nanoscopic structural features of the sieve-like cribellar spigots for capture thread production in the titanoecid spider *N. albofasciata*. The cribellar cuticular surface is covered by thousands of tiny spigots, and this cribellar plate produces non-sticky threads composed of thousands of fines nanofibers. *N. albofasciata* cribellar spigots are typically about 10 μm long, and each spigot has five distinct segments, which is a defining characteristic of this spider. Each cribellar spigot appears as a long individual shaft with a pagoda-like tiered tip. This segmented and flexible structure allows spigots to bend individually and join with adjacent spigots to form cribellar prey-capture thread. The expanded intersegmental spaces generate pearling of the cribellar thread and provide supporting points to hold silk fibrils during the hackling process carried out by the calamistra.

## Data Availability

Materials described in the manuscript, including all relevant raw data, will be freely available to any scientist wishing to use them for non-commercial purposes.
